# Predicting osimertinib‐treatment outcomes through *EGFR* mutant‐fraction monitoring in the circulating tumor DNA of *EGFR* T790M‐positive patients with non‐small cell lung cancer (WJOG8815L)

**DOI:** 10.1002/1878-0261.12841

**Published:** 2020-11-17

**Authors:** Kazuko Sakai, Takayuki Takahama, Mototsugu Shimokawa, Koichi Azuma, Masayuki Takeda, Terufumi Kato, Haruko Daga, Isamu Okamoto, Hiroaki Akamatsu, Shunsuke Teraoka, Akira Ono, Tatsuo Ohira, Toshihide Yokoyama, Nobuyuki Yamamoto, Kazuhiko Nakagawa, Kazuto Nishio

**Affiliations:** ^1^ Department of Genome Biology Faculty of Medicine Kindai University Osaka Japan; ^2^ Department of Medical Oncology Faculty of Medicine Kindai University Osaka Japan; ^3^ Department of Biostatistics Yamaguchi University Graduate School of Medicine Yamaguchi Japan; ^4^ Division of Respirology, Neurology, and Rheumatology Department of Internal Medicine Kurume University School of Medicine Fukuoka Japan; ^5^ Department of Thoracic Oncology Kanagawa Cancer Center Yokohama Japan; ^6^ Department of Medical Oncology Osaka City General Hospital Osaka Japan; ^7^ Research Institute for Diseases of the Chest Graduate School of Medical Sciences Kyushu University Fukuoka Japan; ^8^ Internal Medicine III Wakayama Medical University Wakayama Japan; ^9^ Division of Thoracic Oncology Shizuoka Cancer Center Shizuoka Japan; ^10^ Department of Surgery Tokyo Medical University Tokyo Japan; ^11^ Department of Respiratory Medicine Kurashiki Central Hospital Okayama Japan

**Keywords:** circulating tumor DNA, digital PCR, *EGFR* mutation, liquid biopsy, mutant allele frequency, next‐generation sequencer, osimertinib

## Abstract

The WJOG8815L phase II clinical study involves patients with non‐small cell lung cancer (NSCLC) that harbored the *EGFR* T790M mutation, which confers resistance to EGFR tyrosine kinase inhibitors (TKIs). The purpose of this study was to assess the predictive value of monitoring *EGFR* genomic alterations in circulating tumor DNA (ctDNA) from patients with NSCLC that undergo treatment with the third‐generation EGFR‐TKI osimertinib. Plasma samples of 52 patients harboring the *EGFR* T790M mutation were obtained pretreatment (Pre), on day 1 of treatment cycle 4 (C4) or cycle 9 (C9), and at diagnosis of disease progression or treatment discontinuation (PD/stop). CtDNA was screened for EGFR‐TKI‐sensitizing mutations, the *EGFR* T790M mutation, and other genomic alterations using the cobas *EGFR* Mutation Test v2 (cobas), droplet digital PCR (ddPCR), and targeted deep sequencing. Analysis of the sensitizing—and T790M—*EGFR* mutant fractions (MFs) was used to determine tumor mutational burden. Both MFs were found to decrease during treatment, whereas rebound of the sensitizing *EGFR* MF was observed at PD/stop, suggesting that osimertinib targeted both T790M mutation‐positive tumors and tumors with sensitizing *EGFR* mutations. Significant differences in the response rates and progression‐free survival were observed between the sensitizing *EGFR* MF‐high and sensitizing *EGFR* MF‐low groups (cutoff: median) at C4. In conclusion, ctDNA monitoring for sensitizing *EGFR* mutations at C4 is suitable for predicting the treatment outcomes in NSCLC patients receiving osimertinib (Clinical Trial Registration No.: UMIN000022076).

## Graphical abstract

In the WJOG8815L phase II clinical study, we monitored *EGFR* mutation fractions (MFs) in the circulating tumor DNA (ctDNA) of *EGFR* T790M mutation‐positive patients with non‐small cell lung cancer (NSCLC) that received treatment with the tyrosine kinase inhibitor (TKI) osimertinib. The MF values of TKI‐sensitizing *EGFR* mutations in ctDNA at day 1 of cycle 4 of osimertinib treatment could predict osimertinib treatment outcomes.

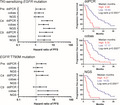

**Abbreviations**
CIs: confidence intervals
ctDNA: circulating tumor DNA
ddPCR: droplet digital PCR
EGFR: epidermal growth factor receptor
MFs: mutant fractions
NGS: next‐generation sequencing
NSCLC: non‐small cell lung cancer
ORR: overall response rate
OS: overall survival
PD: progressive disease
PFS: progression‐free survival
PR: partial response
SD: stable disease
TKI: tyrosine kinase inhibitor

## 1 Introduction

Epidermal growth factor receptor (EGFR) tyrosine kinase inhibitors (TKIs) have been demonstrated to show efficacy in non‐small cell lung cancer (NSCLC) patients with sensitizing *EGFR* mutations [1, 2]. At present, first‐ to third‐generation EGFR‐TKIs serve as standard first‐line treatment for these patients. On the other hand, the emergence of acquired resistance to TKIs often interrupts continued treatment. A number of mechanisms of acquired resistance to EGFR‐TKIs have been described, including the emergence of a secondary *EGFR* T790M mutation in exon 20 and *MET* gene amplification [3]. The T790M *EGFR* mutation is the most frequent cause for acquired resistance to first‐ or second‐generation EGFR‐TKIs and can be detected in up to 50% of patients receiving treatment with these drugs.

Osimertinib is an irreversible T790M‐targeted third‐generation EGFR‐TKI capable of overcoming T790M‐mediated resistance. Its efficacy was evaluated in a clinical phase III study (AURA3) that compared osimertinib with platinum‐based chemotherapy in *EGFR* T790M mutation‐positive patients with advanced NSCLC who had shown progressive disease (PD) after previous EGFR‐TKI treatment [4]. If one were to assess the tumor resistance mechanisms in NSCLC patients receiving treatment with EGFR‐TKIs, a repeat biopsy (re‐biopsy) would be necessary. However, the conventional invasive biopsy procedures can be challenging because of several reasons, including comorbidities [5]. In this context, liquid biopsy of circulating tumor DNA (ctDNA) in the plasma is promising, because it is minimally invasive for genomic analysis. The cobas (cobas EGFR Mutation Test ver 2; Roche Molecular Systems, Pleasanton, CA, USA) is the only ctDNA‐based EGFR mutation assay that is currently approved in Japan, as well as other countries/regions, for the detection of *EGFR* resistance mutations in patients with NSCLC.

The development of sensitive massive parallel sequencing with a next‐generation sequencing (NGS) platform has made it possible to identify gene alterations in ctDNA extracted from plasma samples [6, 7]. The efficacy of osimertinib has been retrospectively evaluated in patients with the *EGFR* T790M mutation detected in plasma ctDNA by several assays, such as droplet digital PCR (ddPCR) analysis and cobas [6, 7, 8]. The NGS platform allows investigation of a panel of 50 cancer‐related genes. Couraud *et al*. [9] reported an assay specificity of 86–100% and sensitivity of 58% for the detection of all 50 genes. Thus, liquid biopsy offers a promising alternative to tissue biopsy for genomic analysis and has been applied in clinical practice for detecting TKI‐sensitizing *EGFR* mutations.

Another advantage of analyzing ctDNA is that it can be used for genomic monitoring throughout the course of disease, from cancer detection to monitoring of the response to therapy, and to understand the mechanisms of resistance. It has been suggested that monitoring of plasma ctDNA for TKI‐sensitizing *EGFR* mutations may be useful for early prediction of the efficacy of EGFR‐TKIs; however, this still remains to be established [10, 11].

We previously reported the safety and efficacy of osimertinib in patients with T790M mutation in ctDNA in the WJOG8815L/LPS study [12]. In this study, a total of 74 patients were identified as being positive for the T790M mutation in the plasma. The overall response rate (ORR) in the evaluable patients positive for the T790M mutation in the ctDNA by the cobas assay (*n* = 49) was 55.1% [95% confidence interval (CI), 40.2–69.3]. The median progression‐free survival (PFS) in the evaluable patients (*n* = 52) was 8.3 months (95% CI, 6.9–12.6 months). Thus, osimertinib is active for T790M‐positive NSCLC. However, mechanisms of resistance to osimertinib, such as *EGFR* C797S and *MET* amplification, and HER2 mutations have already been identified [13].

In this study (WJOG8815L/LPS), conducted as an exploratory biomarker study, we evaluated the usefulness of longitudinal monitoring of ctDNA in *EGFR* T790M mutation‐positive NSCLC patients receiving treatment with osimertinib. Plasma samples were longitudinally collected from the patients during EGFR‐TKI treatment until treatment failure (disease progression); the mutation profile in the plasma ctDNA was analyzed by cobas, ddPCR, and targeted deep sequencing using NGS, in order to investigate the correlation of the mutation profiles with the clinical outcomes.

## 2 Materials and methods

### 2.1 Patients and samples

Patients evaluated in this study were part of the WJOG8815L study which has been described in detail previously [12]. In brief, patients with the *EGFR* T790M mutation detected by cobas or ddPCR (QX100 Droplet Digital PCR System; Bio‐Rad, Hercules, CA, USA) in plasma specimens collected after confirmation of disease progression during treatment with first‐ or second‐generation EGFR‐TKIs received osimertinib until disease progression or treatment discontinuation. The primary endpoint was the ORR, defined as the proportion of patients with measurable regions who showed a complete response (CR) or partial response (PR) that was certified by a second scan after at least 4 weeks by RECIST version 1.1. Secondary endpoints included PFS and overall survival (OS). PFS was defined as the time from administration of the first dose of osimertinib until disease progression or death, regardless of whether the patient discontinued osimertinib treatment or received another therapy before progression. OS was defined as the period from the commencement of osimertinib until death resulting from any cause.

Blood samples (18 mL) were collected in EDTA tubes before the first cycle of treatment (pretreatment, Pre), day 1 of the fourth cycle of treatment (C4), and day 1 of the ninth cycle of treatment (C9), as well as at the PD or treatment discontinuation (stop). Plasma was separated from blood by centrifugation at 1400 ***g*** for 10 min at room temperature and stored at −80 °C until the analyses. ctDNA was isolated using the cfDNA Sample Preparation Kit (Roche Molecular Systems).

The study was performed in accordance with the Declaration of Helsinki, with the approval of the institutional review board of each institution. Written informed consent was obtained from each of the patients. This study has been registered with Clinical Trials Registry (University Medical Hospital Information Network under the identifier 000022076).

### 2.2 cobas EGFR Mutation Test v2

EGFR mutation testing using cobas was performed according to the manufacturer's protocol. Mutation detection was achieved through PCR analysis with the cobas® z480 analyzer (Roche Molecular Systems).

### 2.3 Droplet digital PCR

Droplet digital PCR was carried out as reported previously [6, 7, 14]. The QX100 Droplet Digital PCR System (Bio‐Rad) was used to detect the TKI‐sensitizing *EGFR* mutations, *EGFR* T790M and *EGFR* C797S. The primers and probe for detection of the TKI‐sensitizing *EGFR* mutation and *EGFR* T790M mutation were obtained from Bio‐Rad. The primers and probe reported by Thress *et al*. [15] were used for detection of the *EGFR* C797S mutation. The digital PCR data were analyzed using the quantasoft analytical software package (Bio‐Rad).

### 2.4 NGS analysis

The ctDNA samples were analyzed with NGS panels (Colon and Lung Cancer Research Panel; Thermo Fisher Scientific, Wilmington, DE, USA) for 22 mutation detections. Library preparation and sequencing were performed as described in Ohira *et al*. [16]. Reads were aligned with the hg19 human reference genome, and potential mutations were called using Variant Caller ver. 5.10. For the detection of ctDNA mutations, mutation calling was performed as described previously [16].

### 2.5 Statistical analysis

The mutant fractions (MFs) determined by cobas, ddPCR, and NGS were expressed as units, copies/μL, and mutant allele frequency (%), respectively. The chi‐square test was used to determine the relationships between the patient characteristics and the MFs of TKI‐sensitizing *EGFR* and T790M mutations in the ctDNA obtained at each point. Distribution of PFS was estimated using Kaplan–Meier methods and compared with log‐rank tests. Univariate analysis and multivariate Cox proportional hazards regression models with backward elimination method were performed to assess the potential predictive parameters for PFS. Harrell's concordance index (*c*‐index) was used to evaluate accuracy of prediction for survival analysis [17]. All statistical analyses were performed using jmp version 14.2 and sas version 9.4 (SAS Institute, Cary, NC, USA).

## 3 Results

### 3.1 Patient population

**Fig. 1:**
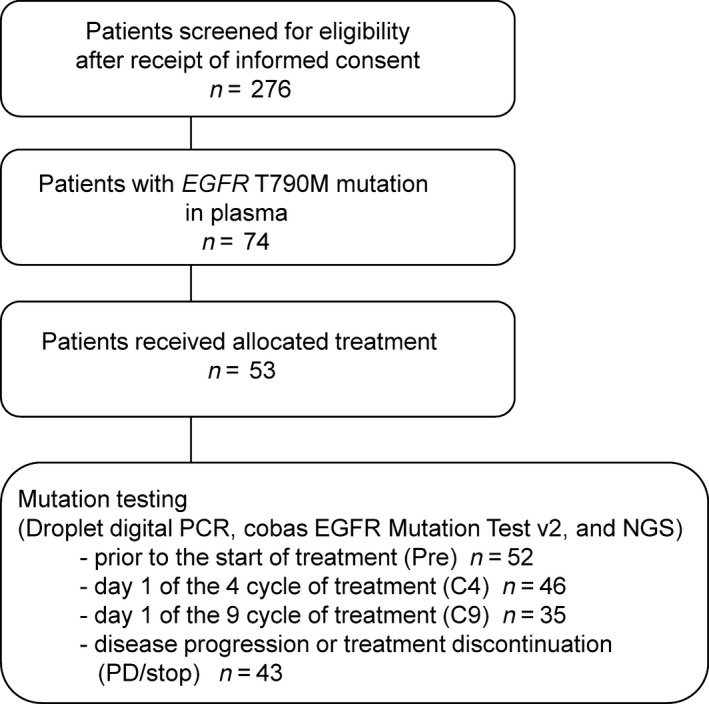
Study flow diagram. Mutation status was determined by an analysis of plasma with the cobas EGFR Mutation Test v2, ddPCR, and NGS. ddPCR, droplet digital polymerase chain reaction (Colon and Lung Cancer Research Panel).

Between June 2016 and November 2017, plasma samples of 276 NSCLC patients at 22 sites across Japan (WJOG8815LPS) were screened by cobas and ddPCR for the presence of *EGFR* T790M mutation in their plasma samples. *EGFR* T790M was detected in seventy‐four patients, and a total of 52 of these 74 patients were enrolled in WJOG8815L study and treated with osimertinib [12]. Plasma samples from the 52 patients were obtained prior to the start of treatment (Pre), day 1 of C4, day 1 of C9, and at disease progression or treatment discontinuation (PD/stop) (*n* = 52, 46, 35, and 43, respectively) (Fig. 1). Baseline patient demographics and clinical characteristics are summarized in Table 1. All of the 52 patients had developed PD during the previous EGFR‐TKI treatment, and 16 patients (30.2%) received at least two prior chemotherapy regimens, including cytotoxic or EGFR‐TKIs [12].

**Table 1:** Characteristics of the enrolled patients.

Characteristics	*n* = 52 (%)
Age	
Median (range)	67 (37–82)
Gender	
Male	17 (32.7)
Female	35 (67.3)
PS	
0	12 (23.1)
1	40 (76.9)
Smoking history	
None	35 (67.3)
Former	17 (32.7)
Genotype of actionable *EGFR* mutation	
Exon 19 deletion	33 (63.5)
L858R	19 (36.5)

### 3.2 Experimental condition of three mutation assays

Circulating tumor DNA was eluted from a fixed plasma volume; thus, to maximize ctDNA, maximum possible input volumes were used for each assay. Median input DNA was 4.98 ng (range: 0.98–49.63) for ddPCR, 8.90 ng (range: 1.76–88.63) for cobas and 2.13 ng (range: 0.42–21.27) for NGS, respectively. As an assay control for ddPCR, the total copy number of *EGFR* was calculated. Median copy number in ddPCR was 991 (range: 266–55 880) for TKI‐sensitizing *EGFR* mutation and 922 (range: 260–41 500) for *EGFR* T790M, respectively. No errors were detected in any of the samples examined by the cobas assay. As an assay control for NGS, on‐target rate (median: 95.2%, range: 81.3–97.2%) and mean depth (median: 21 895, range: 4000–93 746) were calculated (Table S1). None of the three assays had failures in determining MFs and that all are valuable assays for ctDNA monitoring if sufficient amount of ctDNA is available.

### 3.3 Mutation burden, determined as the MF, of TKI‐sensitizing EGFR mutations

Analysis of the plasma ctDNA for TKI‐sensitizing *EGFR* mutations (exon 19 deletion and L858R) was conducted by cobas, ddPCR, and NGS. The TKI‐sensitizing *EGFR* mutation burden quantified as the MF by cobas, ddPCR, and NGS was expressed in units, copies/μL, and mutant allele frequency (%), respectively. The samples were obtained at Pre, C4, C9, and PD/stop time points (Table 2). The median MF values of TKI‐sensitizing *EGFR* mutations determined by ddPCR, cobas, and NGS at Pre were 3.3, 14.3, and 10.0, respectively. The median MF value at C4 and C9 was 0. The MF values at PD/stop determined by the three methods were 0.64 (19.4% compared with the value at Pre), 10.5 (73.4%), and 1.2 (12.0%), respectively. These results indicate that the TKI‐sensitizing *EGFR* mutations in the ctDNA were detectable by all the assays prior to the initiation of osimertinib treatment. Dramatic decrease in the MFs of the TKI‐sensitizing *EGFR* mutations was observed during osimertinib treatment (at C4 and C9), but rebound of the MF (TKI‐sensitizing mutations), although not to the Pre level, was observed at PD/stop (Fig. S1).

**Table 2:** Mutation burden, determined as the MF, of TKI‐sensitizing *EGFR* mutation.

	*n*	Median (range)^a^
ddPCR		
Pre	52	3.3 (0–873.0)
C4	46	0 (0–23.0)
C9	35	0 (0–28.9)
PD/stop	43	0.6 (0–1129.5)
cobas		
Pre	52	14.3 (5.8–22.9)
C4	46	0 (0–19.0)
C9	35	0 (0–18.2)
PD/stop	43	10.5 (0–23.5)
NGS		
Pre	52	10.0 (0–91.2)
C4	46	0 (0–40.7)
C9	35	0 (0–28.7)
PD/stop	43	1.2 (0–81.4)

^a^ MF; ddPCR, copies/µL; cobas, unit; NGS, allele frequency (%).

### 3.4 Mutation burden, determined as the MF, of the EGFR T790M mutation

The MFs of *EGFR* T790M in the ctDNA were also monitored. The median MF values at Pre, determined by ddPCR, cobas, and NGS, were 0.8, 9.4, and 2.7, respectively (Table 3). On the other hand, the median MF value was 0, as determined by all the assays, at C4, C9, and PD/stop. This result suggests that the T790M‐positive tumor clones decreased during osimertinib treatment, and no dramatic regrowth of T790M‐positive tumor was observed even at PD/stop.

**Table 3:** Mutation burden, determined as the MF, of the *EGFR* T790M mutation.

	Median (range)^a^
ddPCR	
Pre	0.8 (0–511.5)
C4	0 (0–0.1)
C9	0 (0–15.9)
PD/stop	0 (0–652.0)
cobas	
Pre	9.4 (0–20.0)
C4	0 (–)
C9	0 (0–13.5)
PD/stop	0 (0–19.3)
NGS	
Pre	2.7 (0–72.8)
C4	0 (0–0.5)
C9	0 (0–13.7)
PD/stop	0 (0–58.2)

^a^ MF; ddPCR, copies/µL; cobas, unit; NGS, allele frequency (%).

### 3.5 Association between the MF and response to osimertinib

The median MF values of TKI‐sensitizing *EGFR* mutations in the ctDNA were used as the cutoff points to categorize the patients into MF‐high and MF‐low groups. The response rates [PR or non‐response (including SD or PD)] were compared between the MF‐high and MF‐low groups using the chi‐squared test, based on our hypothesis that monitoring of *EGFR* mutant alleles in the ctDNA could be correlated with the clinical outcome. No significant differences in the response rates were observed between the MF‐high and MF‐low groups for TKI‐sensitizing *EGFR* mutations at Pre and C9 (Table 4). On the other hand, a significant difference in the response rates between the two groups (classified according to the median MF values determined by the three assays) was observed at C4. The response rates at C4 of the MF‐high and MF‐low groups determined by ddPCR were 20.7% and 79.3%, respectively. The corresponding rates at C4 in the groups determined by cobas were 31.0% and 69.0%, respectively, and in the two groups classified according to the median MF value determined by NGS were 13.8% and 86.2%, respectively. On the other hand, no significant differences in the response rates between the MF‐high and MF‐low groups for the T790M mutation as classified according to the median MF values determined by any of the three methods (Table 5) were observed at any of the sampling points (Pre, C4, and C9). These results suggest that ctDNA monitoring for TKI‐sensitizing *EGFR* mutations at C4 was strongly associated with tumor response.

**Table 4:** Relationship between the MF of TKI‐sensitizing *EGFR* mutation and tumor response to osimertinib.

Assay	Sampling point	MF	PR (*n* = 29)	SD/PD (*n* = 23)	*P* *
ddPCR	Pre	High	12 (41.4)	14 (60.9)	0.1627
		Low	17 (58.6)	9 (39.1)	
	C4	High	6 (20.7)	10 (58.8)	0.0088*
		Low	23 (79.3)	7 (41.2)	
	C9	High	5 (19.2)	3 (33.3)	0.3852
		Low	21 (80.8)	6 (66.7)	
cobas	Pre	High	14 (48.3)	12 (52.2)	0.7801
		Low	15 (51.7)	11 (47.8)	
	C4	High	9 (31.0)	13 (76.5)	0.0029*
		Low	20 (69.0)	4 (23.5)	
	C9	High	7 (26.9)	5 (55.6)	0.1188
		Low	19 (73.1)	4 (44.4)	
NGS	Pre	High	14 (48.3)	12 (52.2)	0.7801
		Low	15 (51.7)	11 (47.8)	
	C4	High	4 (13.8)	9 (52.9)	0.0044*
		Low	25 (86.2)	8 (47.1)	
	C9	High	6 (23.1)	3 (33.3)	0.5440
		Low	20 (76.9)	6 (66.7)	

* *P* < 0.05 (chi‐squared test).

**Table 5:** Relationship between the MF of *EGFR* T790M mutation and tumor response to osimertinib.

Assay	Sampling point	MF	PR (*n* = 29)	SD/PD (*n* = 23)	*P* *
ddPCR	Pre	High	12 (41.4)	14 (60.9)	0.1627
		Low	17 (58.6)	9 (39.1)	
	C4	High	6 (20.7)	3 (17.6)	0.8017
		Low	23 (79.3)	14 (82.4)	
	C9	High	6 (23.1)	2 (22.2)	0.9580
		Low	20 (76.9)	7 (77.8)	
cobas	Pre	High	13 (44.8)	13 (56.5)	0.4022
		Low	16 (55.2)	10 (43.5)	
	C9	High	3 (11.5)	0 (0)	0.2865
		Low	23 (88.5)	9 (100)	
NGS	Pre	High	13 (44.8)	13 (56.5)	0.4022
		Low	16 (55.2)	10 (43.5)	
	C4	High	2 (6.9)	0 (0)	0.2682
		Low	27 (93.1)	17 (100)	
	C9	High	3 (11.5)	0 (0)	0.2865
		Low	23 (88.5)	9 (100)	

* *P* < 0.05 (chi‐squared test).

### 3.6 Association between MFs and the survival time

**Fig. 2:**
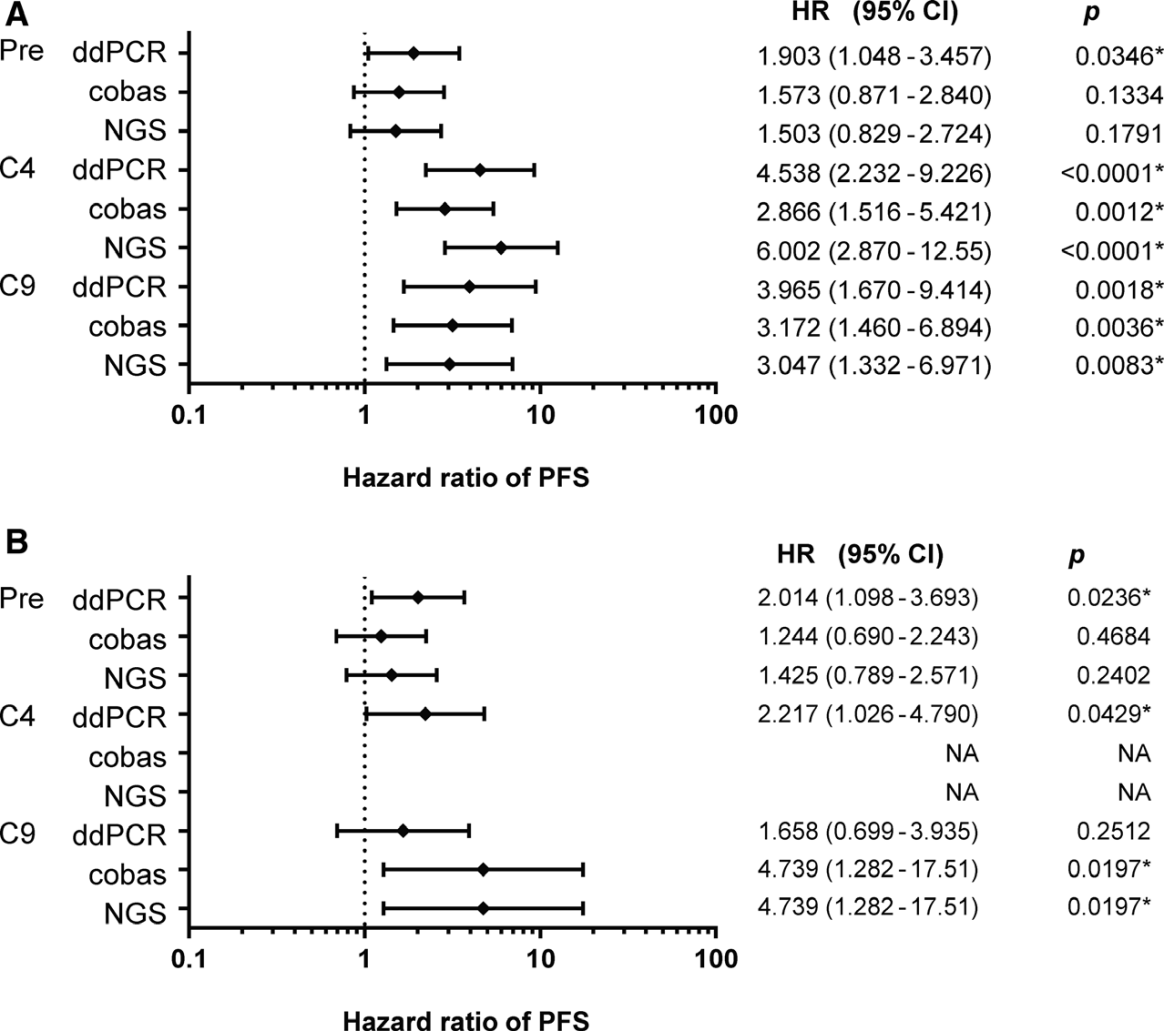
Forest plot based on univariate hazard ratio (HR) for PFS according to the mutation fraction (MF) of *EGFR*‐sensitizing (A) and *EGFR* T790M mutations (B) in ctDNA detected by ddPCR, cobas, or NGS.

To investigate the association of the MF values with the duration of PFS, univariate regression analysis was performed using a Cox proportional hazards model for the MF‐high and MF‐low groups (Fig. 2A). In regard to the MF values for TKI‐sensitizing *EGFR* mutations, significant difference in the PFS was observed between the MF‐high and MF‐low groups classified prior to treatment (Pre) according to the median MF values determined by ddPCR (Fig. S2A) but not cobas or NGS (Fig. S2C,E). On the other hand, significant differences in the PFS were observed between the MF‐high and MF‐low groups classified at C4 and C9 by any of the three assays. In particular, the shortest PFS values were observed in the MF‐high groups (4.94, 6.32, and 4.27 months for ddPCR, cobas, and NGS, respectively) as compared to the MF‐low group (15.92, 17.17, and 14.52 months, respectively) classified at C4 by ddPCR, cobas, and NGS (*P* values according to the log‐rank test, *P* < 0.0001, 0.0007, and < 0.0001, respectively) (Fig. S2B,D,F). The MF‐high group at C4 also showed a significantly shorter OS as compared to the MF‐low group at C4 (data not shown). Differences in the PFS between the MF‐high and MF‐low groups at C9 were also observed when the classification was made according to the median MF values determined by all of the three assays. The OS was also significantly shorter in the MF‐high as compared to the MF‐low group at C9 (data not shown).

In regard to the MF values for the T790M mutation, significant differences in the PFS between the MF‐high and MF‐low groups were observed at Pre or C4 (Fig. 2B) determined by ddPCR. On the other hand, significant difference in the PFS between the MF‐high and MF‐low groups was observed at C9, when the groups were classified according to the median MF values determined by cobas and NGS, but not when ddPCR was used for the classification. These results suggest that MF measurement of TKI‐sensitizing *EGFR* mutations rather than that of the T790M mutation at C4 and C9 could allow the PFS in response to osimertinib treatment be associated.

To evaluate the accuracy and predictability of the assay, the C statistic (concordance index, *c*‐index) was estimated. The *c*‐indexes of TKI‐sensitizing *EGFR* mutations at Pre (for NGS), C4 (for ddPCR and NGS), and C9 (for ddPCR and NGS), and *EGFR* T790M mutation at Pre (for ddPCR), C4 (for ddPCR), and C9 (for ddPCR) were greater than 0.6 (Table 6), indicating a higher accuracy for these parameters.

**Table 6:** Multivariate analyses between *EGFR* mutation fraction and clinicopathological parameters.

	Sampling point	Assay	*c*‐index	Multivariate	
				HR (95% CI)	*P*
TKI‐sensitizing *EGFR* mutation	Pre	ddPCR	0.5802	1.903 (1.048–3.457)	0.0346*
		cobas	0.5459	NS	NS
		NGS	0.6190	NS	NS
	C4	ddPCR	0.6085	4.538 (2.232–9.226)	<0.0001*
		cobas	0.5459	2.866 (1.516–5.421)	0.0012*
		NGS	0.6190	8.850 (3.743–20.921)	<0.0001*
	C9	ddPCR	0.6085	3.965 (1.670–9.414)	0.0018*
		cobas	0.5459	3.172 (1.460–6.894)	0.0036*
		NGS	0.6190	3.047 (1.332–6.971)	0.0083*
*EGFR* T790M mutation	Pre	ddPCR	0.6067	2.014 (1.098–3.693)	0.0236*
		cobas	0.5467	NS	NS
		NGS	0.5697	NS	NS
	C4	ddPCR	0.6067	2.217 (1.026–4.790)	0.0429*
		cobas	0.5467	NA	NA
		NGS	0.5697	NA	NA
	C9	ddPCR	0.6067	NS	NS
		cobas	0.5467	4.739 (1.282–17.51)	0.0197*
		NGS	0.5697	4.739 (1.282–17.51)	0.0197*

Reduced Cox multivariate analysis with stepwise backward elimination. The variables co‐analyzed were *EGFR* mutation fraction in each point and clinicopathologic parameters [age (≥ 65 vs. < 65), gender, PS (0 or 1), smoking history (none vs. former), and actionable *EGFR* mutation status determined before treatment]. Age, gender, PS, smoking history, and genotype of *EGFR* mutation were not significantly correlated with PFS by univariate analysis with HR and *P* values of 0.9240 and 0.7992, 1.119 and 0.7247, 1.8770 and 0.0950, 0.8550 and 0.6295, and 0.6750 and 0.2175, respectively. NA, not available; NS, no significant variables remained.

* *P* < 0.05.

To further determine whether a high MF at C4 could still predict PFS considering additional variables, we performed reduced Cox multivariate analysis with stepwise backward elimination of the least significant until only significant covariables remained. The variables co‐analyzed with each mutation status were clinicopathological parameters [age, gender, performance status (PS), smoking history, and actionable *EGFR* mutation status determined before treatment] (Table 1). There were no significant associations between clinicopathological parameters and PFS by univariate analysis (Table 6), whereas multivariate analysis of *EGFR*‐sensitizing mutations at C4 and C9 was identified as significant covariates by all three assays (Table 6). In addition, high MF at C4 yielded lower *P* values by all assays in the reduced multivariate analysis model compared with C9, thus suggesting that MF at C4 might be a stronger predictor.

### 3.7 Molecular profiling of ctDNA associated with resistance to osimertinib by the NGS platform

In order to understand the multiple mechanisms of resistance to osimertinib, the MF values were compared between PD/stop and other sampling points (Table S2). Increase in the MF values for TKI‐sensitizing *EGFR* mutations at PD/stop was observed in 14.0%, 23.3%, and 18.6% of the ctDNA samples processed by ddPCR, cobas, and NGS, respectively. Increase in the MF values for the T790M mutation was observed in 7.0%, 9.3%, and 14.0% of the samples processed by the three methods, respectively, but increases in the MF values for the T790M mutation at PD/stop were smaller than the increases in the MF values for the TKI‐sensitizing *EGFR* mutations. These results suggest that the MF value for TKI‐sensitizing *EGFR* mutations reflects the tumor burden more reliably than that for the *EGFR* T790M mutation.

We, as also other researchers, have previously reported that NGS using multitargeted panels is useful for molecular profiling of ctDNA in NSCLC patients treated with EGFR‐TKIs. Several mutated alleles (MF range: 0.1–91.2) were detected in ctDNA by NGS in this study (Table S3). Of the 21 genes evaluated, excluding the *EGFR* gene, mutations in Pre plasma samples were detected for *TP53* (38.5%), *CTNNB1* (5.8%), *PIK3CA* (3.8%), *KRAS* (1.9%), *PTEN* (1.9%), and *SMAD4* (1.9%). After treatment, mutations in *TP53* (5.8%), *PIK3CA* (1.9%), *KRAS* (1.9%), and *BRAF* (1.9%) were detected at C4, and *TP53* (1.9%) and *PIK3CA* (3.8%) at C9, respectively. At treatment discontinuation due to PD, mutations for *TP53* (17.3%), *CTNNB1* (1.9%), *KRAS* (1.9%), *PTEN* (1.9%), *BRAF* (1.9%), and *NRAS* (1.9%) were detected (Fig. S3). In addition, the mutation frequencies of these genes during treatment were comparable to that of *EGFR*‐sensitizing mutations.

Patterns of MFs with mutations other than *EGFR* mutations were also monitored during treatment for the 24 cases. Other than *PIK3CA* mutations, patterns of *TP53*, *PTEN*, *SMAD4*, and *CTNNB1* MFs showed similar trends as the TKI‐sensitizing *EGFR* mutation (Fig. S4).

Newly detected mutations at PD/stop are shown in Table S4. *EGFR* C797S was newly detected at PD/stop in 6/11 (54.8%) cases with T790M‐positive at PD/stop among 11 patients with T790M‐positive at PD/stop. *EGFR* L718Q [18, 19, 20] was also detected at PD/stop in one case. *BRAF* V600E and *NRAS* Q61K were detected at PD/stop. The various mutations mentioned above are considered to be related to acquired resistance to osimertinib.

## 4 Discussion

In this study, we demonstrated that longitudinal disease monitoring by liquid biopsy may be a powerful tool for the detection of disease progression during EGFR‐TKI treatment in NSCLC patients. This was also shown in an exploratory study by Mok and colleagues using plasma ctDNA samples from patients with advanced NSCLC randomized to receive six cycles of gemcitabine and platinum‐based chemotherapy sequenced by either erlotinib or placebo [21]. They demonstrated that mutant *EGFR* allele levels measured in ctDNA at the baseline, after three cycles of treatment, and at the time of diagnosis of disease progression were correlated with the response rates and survival outcomes in a subset of patients. Both PFS and OS were significantly longer in the patients with no detectable mutant *EGFR* alleles in ctDNA compared to patients with detectable mutant *EGFR* alleles in ctDNA after three cycles of treatment. Their study provided evidence to show that monitoring of the changes in the levels of mutant *EGFR* alleles may be useful to predict the benefit of continued treatment with the first‐generation *EGFR*‐TKIs in advanced NSCLC patients. However, no data in relation to treatment with osimertinib were reported from that study.

Focusing *EGFR*‐sensitizing and T790M mutation status before treatment, significant longer PFS was observed in the patients with MF‐low of *EGFR*‐sensitizing and T790M compared with MF‐high patients detected by ddPCR (Fig. 2A,B). On the other hand, no significant difference was observed by cobas nor NGS. Therefore, the predictive value of *EGFR* mutation status in ctDNA is not sufficient in our sample cohort.

Our data from the present study suggest that monitoring of changes in the MFs of TKI‐sensitizing *EGFR* mutations might be useful for predicting the benefit of continued treatment with osimertinib.

However, the changes in the MFs of TKI‐sensitizing *EGFR* mutations could not be calculated in our study, because in most cases, these mutant alleles were not detectable in the ctDNA during treatment (the median value was zero). Our study demonstrated that the ctDNA MFs calculated after 4 cycles of treatment by ddPCR were the most powerful to predict the response to continued treatment and the PFS. The MFs of TKI‐sensitizing *EGFR* mutations after nine cycles of treatment were also useful to predict the PFS, but not the tumor response. It is reasonable to conclude that the tumor response rate was better correlated with the MFs of TKI‐sensitizing *EGFR* mutations determined in the earlier phases of treatment, that is, at C4 vs. C9.

We simultaneously analyzed the MFs of the mutant *EGFR* alleles in the ctDNA by three techniques, namely ddPCR, cobas, and NGS. In our sample cohort, there were no cases of failure of any of the three assays to determine the MFs. The MF values obtained by all three assays at C4 were consistently predictive of the response rate and PFS. Our previous study demonstrated that amplicon‐based NGS can detect the *EGFR* T790M mutation in ctDNA and NGS using the molecular barcoding technology [7]. Recently, ultrasensitive NGS assays have become available for ctDNA analysis [22, 23]. However, it is considered that each of real‐time PCR (cobas), ddPCR, and conventional NGS is sufficient for ctDNA monitoring, if a sufficient amount of ctDNA is available.

Our study demonstrated MFs of TKI‐sensitizing *EGFR* mutations are a better predictive marker than that of the *EGFR* T790M mutation. This is easy to understand, because the main target of osimertinib is the mutational protein encoded by *EGFR* T790M, and osimertinib is active clinically against T790M mutation‐positive NSCLC [4]. Our data provide proof for the notion that osimertinib targets T790M‐positive tumors and decreases T790M‐positive clones. Analysis of the MFs of TKI‐sensitizing mutations and the T790M mutation at PD in our study (Tables 2 and 3) revealed the re‐appearance of TKI‐sensitizing *EGFR* mutations, but not of the T790M mutation. These data suggest the existence of new alternative resistant mechanisms for acquisition of resistance to osimertinib. In an exploratory analysis, plasma genotyping of seven *EGFR* mutant patients who were enrolled in the phase I AURA study and developed acquired resistance to osimertinib therapy revealed an *EGFR* C797S resistance mutation in one of the patients. A subsequent analysis by ddPCR assay of serial plasma samples from 15 patients treated with osimertinib revealed the presence of an acquired C797S resistance mutation in six of these patients, highlighting the importance of these mechanisms of acquired resistance to osimertinib [15]. In our study, *EGFR* C797S and T790M were detected at PD/stop in 6/11 (54.8%) of the patients together. C797S mutation is known to contribute to osimertinib resistance in the Japanese population [24]. Other possible resistance‐related genotypes such as *BRAF* V600E [25] and *NRAS* Q61K [25, 26] in ctDNA were also detected by NGS at PD/stop in this study. These mutations are actionable for activation of bypass pathways such as BRAF and MAPK signaling pathways. *EGFR* L718Q was also detected at PD/stop in one patient. The *EGFR* L718 mutation, detected in the absence of the T790M mutation, has been reported as a mutation conferring resistance to osimertinib [18, 19, 20]. The patient in whom the *EGFR* L718 mutation was detected at PD/stop in our study also showed the T790M mutation, and further study is necessary to elucidate the significance of this genotype. Clarification of the specific mechanisms of acquisition of resistance to osimertinib during treatment in each patient could have a significant clinical impact. In this context, multiplexed technologies, including the NGS technology, will be particularly important.

We previously reported that copy number alterations of *MET* and *HER2* copy number gain in the ctDNA were detectable after *EGFR*‐TKI treatment by ddPCR and NGS [7]. However, in our present study cohort, no gene copy alterations in the ctDNA were detected by NGS at any of the sampling time points. The low sensitivity of NGS to copy number alterations could explain this result.

Thus, employing plasma genotyping is particularly pertinent to targeted therapy, largely because assaying ctDNA allows for a noninvasive genomic monitoring approach for predicting the clinical outcomes and also detecting resistance mechanisms in real time.

## 5 Conclusion

Overall, we have demonstrated that the mutation status of cancer‐related genes can be monitored in the ctDNA by cobas, ddPCR, and NGS. The MF values of TKI‐sensitizing *EGFR* mutations in ctDNA at the onset of C4 of treatment might be useful to predict the clinical outcomes of patients receiving treatment with osimertinib; however, further validation studies will be needed. In addition, NGS analysis of ctDNA yielded additional data that may be useful in revealing additional mechanisms of resistance to osimertinib.

## Conflict of interest

KS reports personal fees from Roche Diagnostics, Bio‐Rad, SRL Diagnostics, AstraZeneca, and Chugai Pharmaceutical outside the submitted work. TT reports personal fees from AstraZeneca, Roche Diagnostics, and Boehringer Ingelheim during the conduct of the study. MS reports personal fees from Sysmex outside the submitted work. KA reports personal fees from AstraZeneca, MSD, Ono Pharmaceutical, Bristol Myers Squibb, and Chugai Pharmaceutical outside the submitted work. MT reports personal fees from Chugai Pharmaceutical, AstraZeneca, Bristol Myers Squibb, and Novartis Pharma outside the submitted work. TK reports personal fees from AstraZeneca, Boehringer Ingelheim, Chugai Pharmaceutical, Eli Lilly, and Ono Pharmaceutical, and grants from AbbVie, AstraZeneca, Bristol Myers Squibb, Chugai Pharmaceutical, Eli Lilly, Kyorin Pharmaceutical, Kyowa Kirin, Merck Serono, MSD, Novartis, Ono Pharmaceutical, Pfizer, Regeneron Pharmaceuticals, and Taiho Pharmaceutical outside the submitted work. HD reports personal fees from Ono Pharmaceutical, Chugai Pharmaceutical, MSD, and Taiho Pharmaceutical and grants from Ono Pharmaceutical, Chugai Pharmaceutical, AstraZeneca, Pfizer, and Taiho Pharmaceutical outside the submitted work. IO reports grants from Boehringer Ingelheim during the conduct of the study; personal fees from Ono Pharmaceutical, MSD, Eli Lilly, Bristol Myers Squibb, Chugai Pharmaceutical, and Pfizer; and grants from Ono Pharmaceutical, MSD, Eli Lilly, Astellas Pharma, Bristol Myers Squibb, Novartis, Chugai Pharmaceutical, and AbbVie outside the submitted work. HA reports personal fees from AstraZeneca, Boehringer Ingelheim, Chugai Pharmaceutical, and Pfizer during the conduct of the study, and grants from Chugai Pharmaceutical outside the submitted work. ST reports personal fees from AstraZeneca, Chugai Pharmaceutical, Novartis, Taiho Pharmaceutical, Ono Pharmaceutical, Boehringer Ingelheim, and AbbVie outside the submitted work. AO reports personal fees from Taiho Pharmaceutical, Ono Pharmaceutical, Chugai Pharmaceutical, Novartis, AstraZeneca, and MSD outside the submitted work. TY reports personal fees from AstraZeneca, Boehringer Ingelheim, Bristol Myers Squibb, Eli Lilly, Ono Pharmaceutical, Taiho Pharmaceutical, MSD, Pfizer, Chugai Pharmaceutical, and Novartis outside the submitted work. NY reports personal fees from MSD, AstraZeneca, Ono Pharmaceutical, Thermo Fisher Scientific, Daiichi Sankyo, Taiho Pharmaceutical, Takeda Pharmaceutical, Chugai Pharmaceutical, Eli Lilly, Boehringer Ingelheim, Novartis, Pfizer, Bristol Myers Squibb, Nippon Kayaku, and Life Technologies Japan, and grants from MSD, AstraZeneca, Ono Pharmaceutical, Daiichi Sankyo, Taiho Pharmaceutical, Chugai Pharmaceutical, Eli Lilly, Boehringer Ingelheim, Novartis, Pfizer, Astellas, AbbVie, Kyorin Pharmaceutical, Shionogi, Tsumura, Astellas Amgen BioPharma, Terumo, Toppan Printing, and Tosoh outside the submitted work. KN reports grants and personal fees from AstraZeneca, Astellas, MSD, Ono Pharmaceutical, Boehringer Ingelheim, Novartis, Pfizer, Bristol Myers Squibb, Eli Lilly, Chugai Pharmaceutical, Daiichi Sankyo, and Merck Serono/Merck BioPharma during the conduct of the study; personal fees from Clinical Trial Co., Medicus Shuppan, Care Net, Inc, Reno. Medical, Kyorin Pharmaceutical, Medical Review, Roche Diagnostics, Bayer Yakuhin, Medical Mobile Communications, 3H Clinical Trial Inc., Nichi‐Iko Pharmaceutical, Takeda Pharmaceutical, Taiho Pharmaceutical, SymBio Pharmaceuticals, Nanzando, Yodosha, Nikkei Business Publications, Thermo Fisher Scientific, Yomiuri Telecasting Corp., Nippon Kayaku, and AbbVie, and grants from Takeda Pharmaceutical, Taiho Pharmaceutical, SymBio Pharmaceuticals, AbbVie, inVentiv Health Japan, ICON Japan, Gritstone Oncology, Parexel International, Kissei Pharmaceutical, EPS Corporation, Syneos Health, Pfizer R&D Japan, A2 Healthcare Corp., Quintiles/IQVIA Services Japan, EP‐CRSU, Linical, Eisai, CMIC Shift Zero, Kyowa Hakko Kirin, Bayer Yakuhin, EPS International, and Otsuka Pharmaceutical outside the submitted work. KN reports personal fees from Otsuka Pharmaceutical, Life Technologies Japan, Boehringer Ingelheim, Eli Lilly, Chugai Pharmaceutical, Eisai, Pfizer, Novartis, MSD, Ono Pharmaceutical, Bristol Myers Squibb, SymBio Pharmaceuticals, Solasia Pharma, Yakult Honsha, Roche Diagnostics, AstraZeneca, Sanofi, Guardant Health, Takeda Pharmaceutical, and Kobayashi Pharmaceutical and grants from Otsuka Pharmaceutical, Life Technologies Japan, Boehringer Ingelheim, Eli Lilly, Ignyta, and Astellas Pharma outside the submitted work. TO has no conflicts of interest.

## Author contributions

KS, TT, NY, KN, and KN designed the study. TT, MS, KA, MT, TK, HD, IO, HA, ST, AO, TO, and TY collected the data and reviewed the manuscript. KS, TT, MS, and KN analyzed the data. KS and KN wrote the article. All the authors approved the final manuscript.

## Supporting information


**Fig. S1.** The time‐course of the MFs of *EGFR* TKI‐sensitizing and T790M mutations in the 52 patients.

**Fig. S2.** Kaplan–Meier analysis demonstrating the PFS in the patients stratified into MF‐high and MF‐low groups by the median MF value.

**Fig. S3.** Frequency of gene mutations detected by next generation sequencing (Colon and Lung Cancer Research Panel for 22 genes).

**Fig. S4.** Changes of the mutation fraction for gene mutations detected by NGS.

**Table S1.** Detailed summary of cross‐platform temporal mutation detection.

**Table S2.** Mutations whose MFs increased at PD/stop.

**Table S3.** Detected mutations by NGS.

**Table S4.** Newly detected mutations at PD/stop.
