# Supplementary figures and images for: Predicting osimertinib‐treatment outcomes through EGFR mutant‐fraction monitoring in the circulating tumor DNA of EGFR T790M‐positive patients with non‐small cell lung cancer (WJOG8815L)
Source: Mol Oncol. 2020 Nov 17;15(1):126–37. doi: 10.1002/1878-0261.12841 (PMC7782093; doi:10.1002/1878-0261.12841)

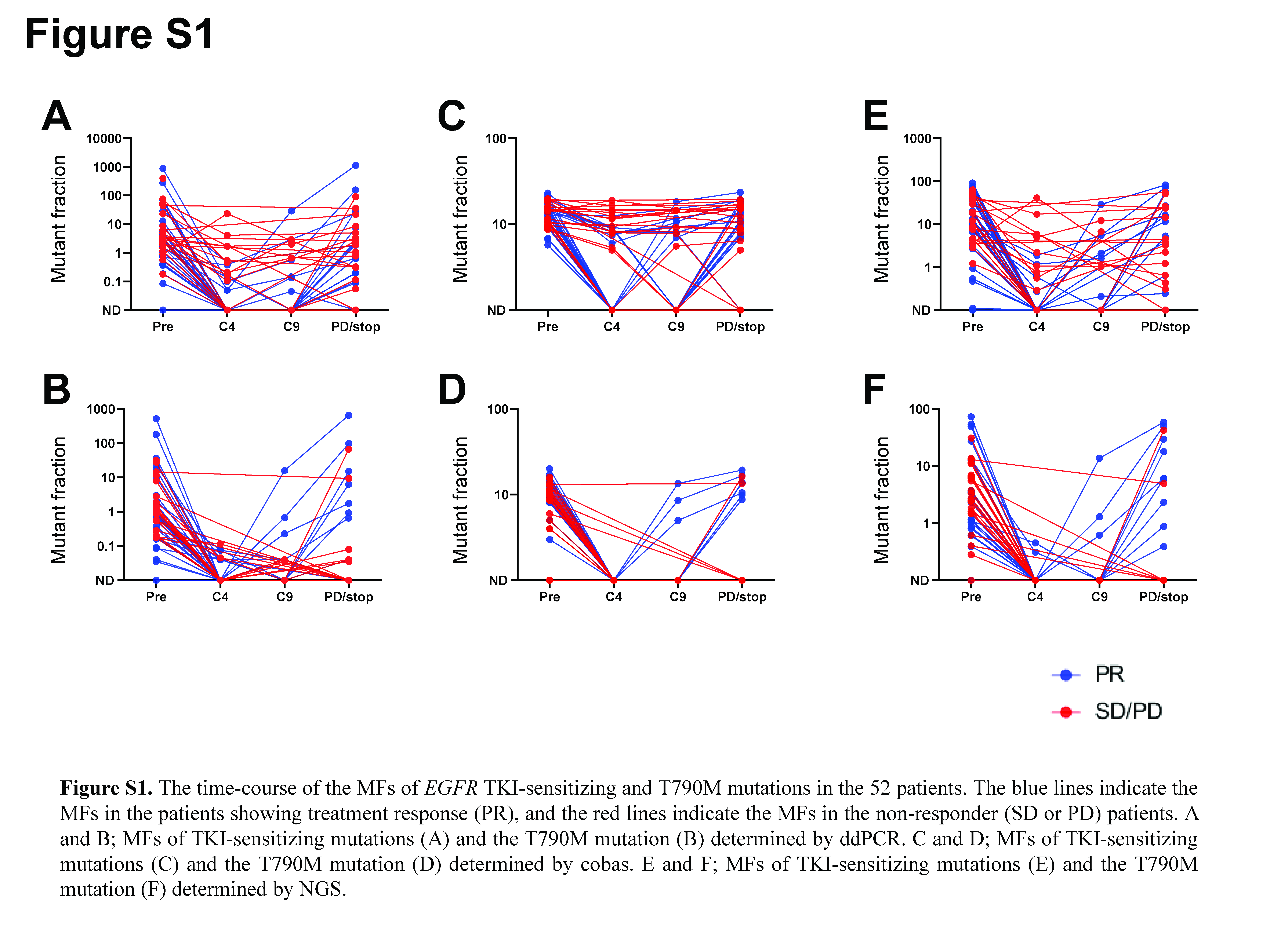

Supplement: Supplementary file 1 — Fig. S1. The time‐course of the MFs of EGFR TKI‐sensitizing and T790M mutations in the 52 patients. [file MOL2-15-126-s001.tiff]

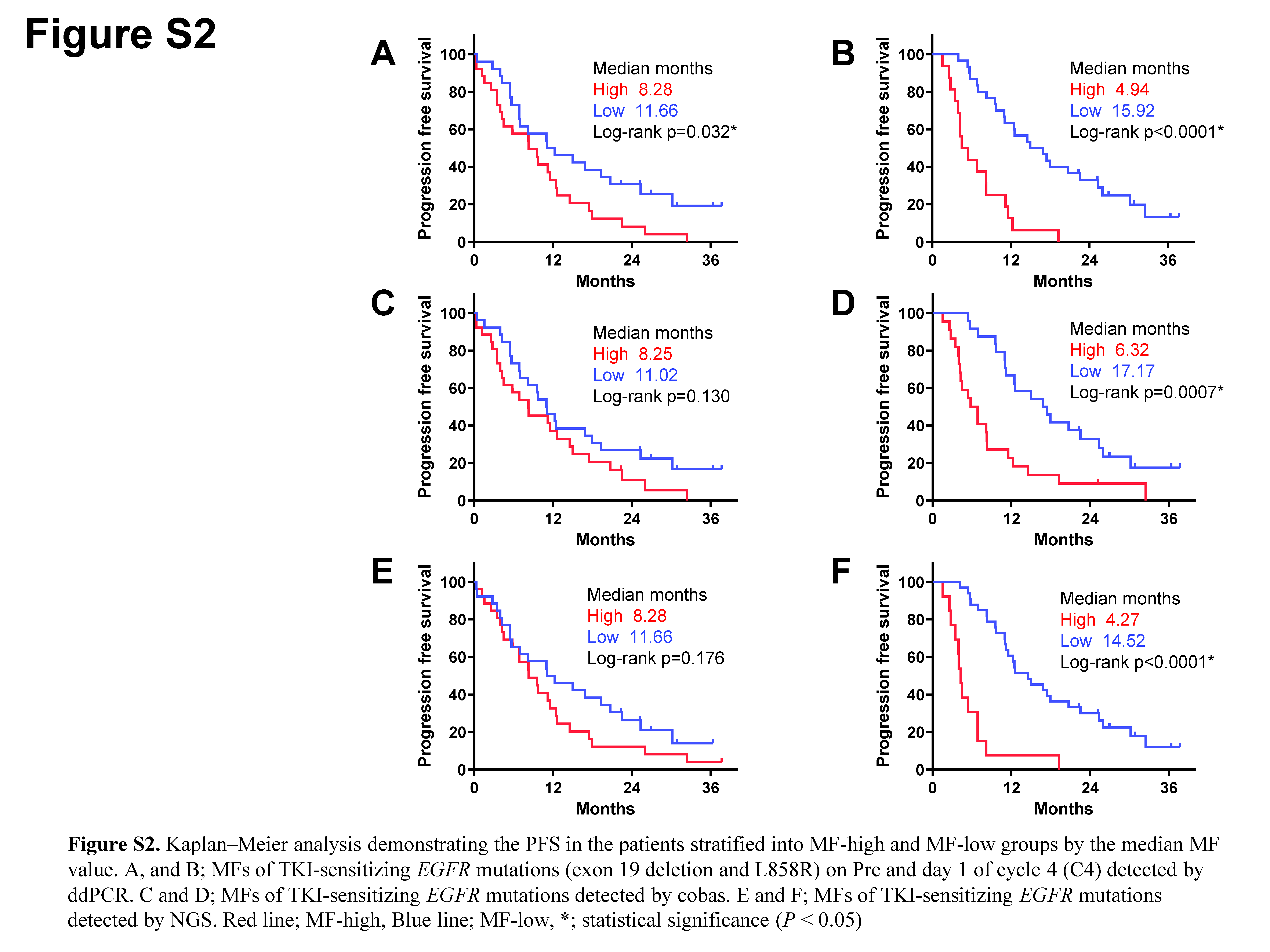

Supplement: Supplementary file 2 — Fig. S2. Kaplan–Meier analysis demonstrating the PFS in the patients stratified into MF‐high and MF‐low groups by the median MF value. [file MOL2-15-126-s002.tiff]

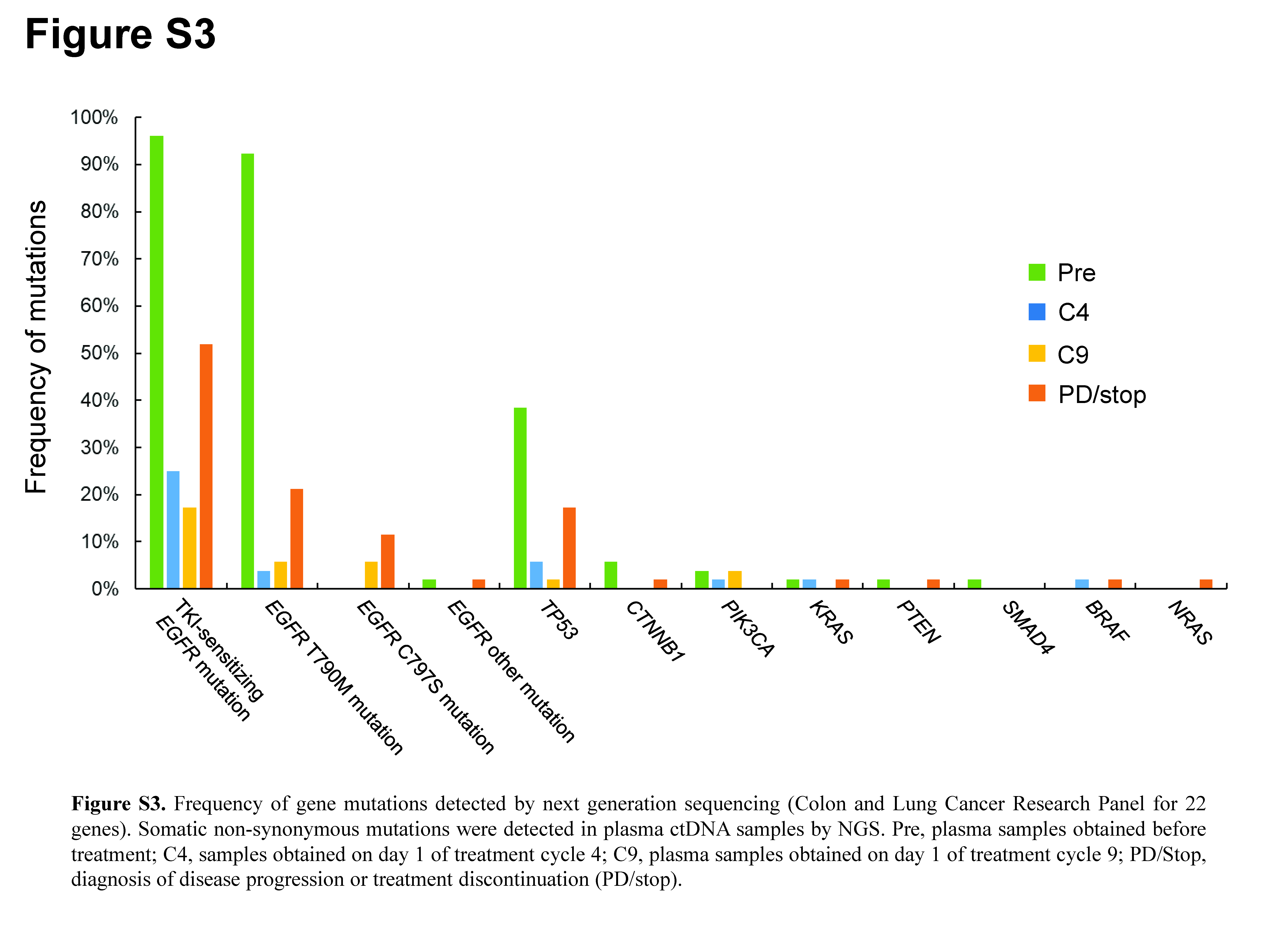

Supplement: Supplementary file 3 — Fig. S3. Frequency of gene mutations detected by next generation sequencing (Colon and Lung Cancer Research Panel for 22 genes). [file MOL2-15-126-s003.tiff]

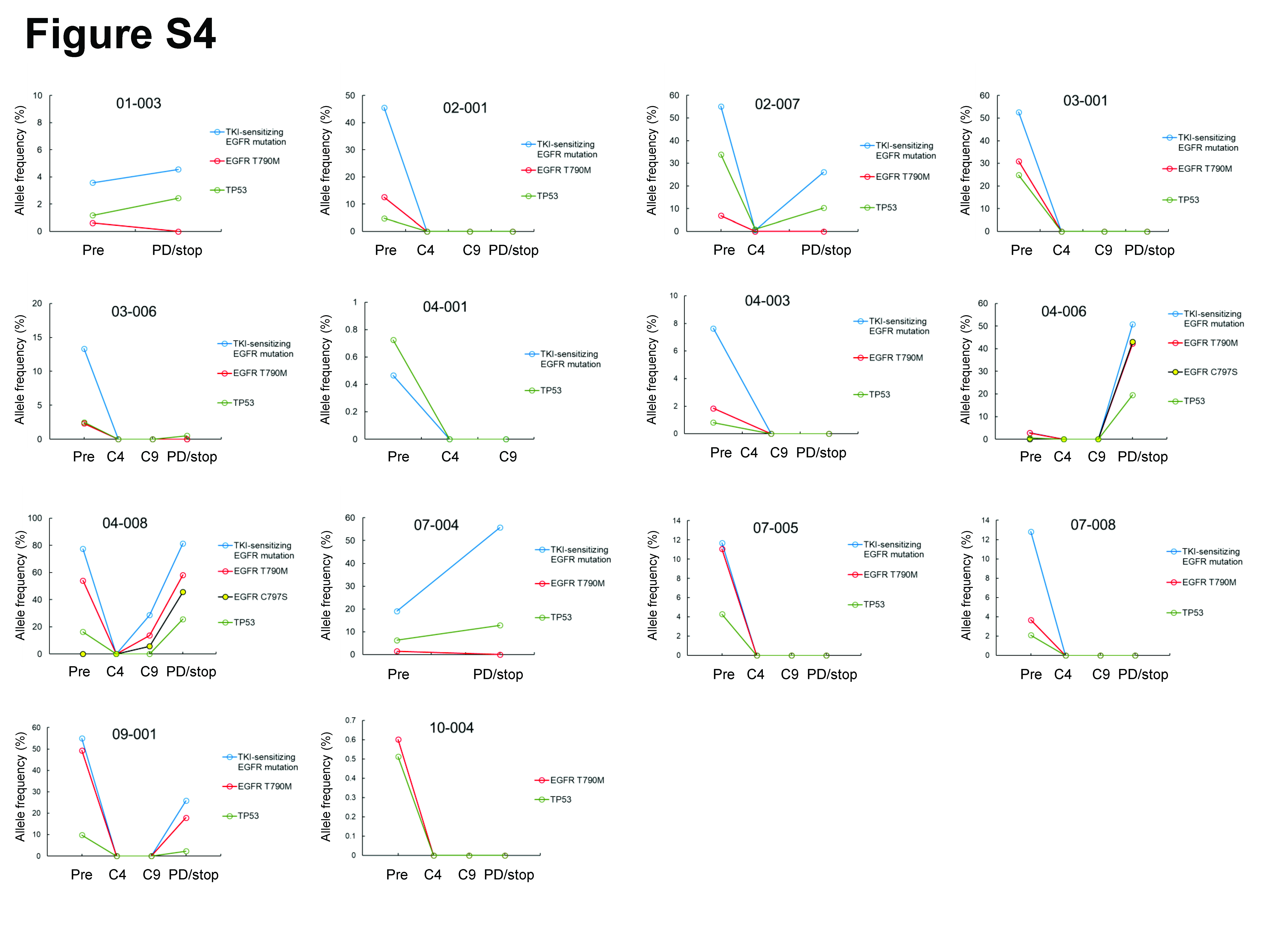

Supplement: Supplementary file 4 — Fig. S4. Changes of the mutation fraction for gene mutations detected by NGS. [file MOL2-15-126-s004.zip › mol212841-sup-0004-FigS4-1.tiff]

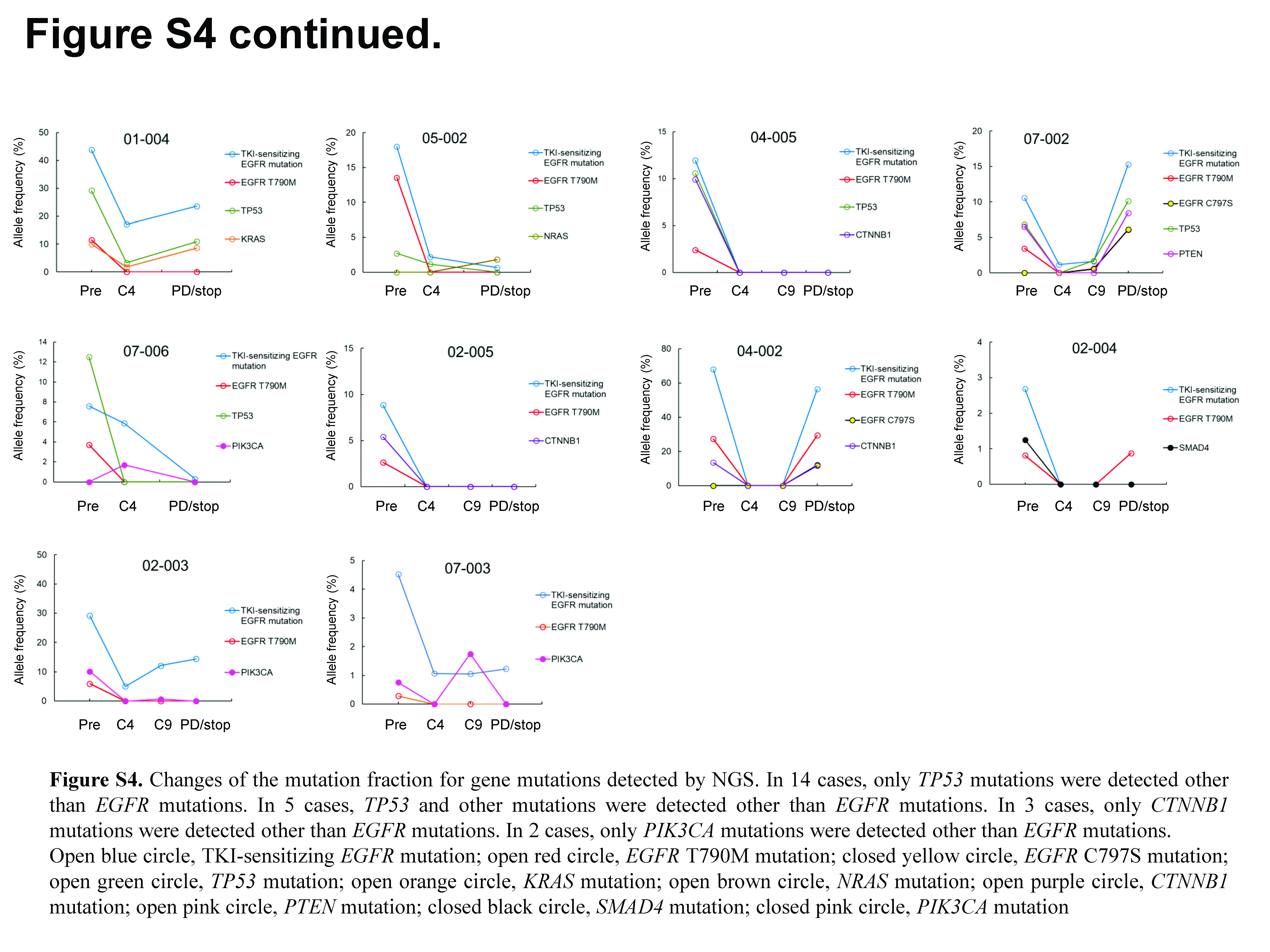

Supplement: Supplementary file 4 — Fig. S4. Changes of the mutation fraction for gene mutations detected by NGS. [file MOL2-15-126-s004.zip › mol212841-sup-0005-FigS4-2.tiff]
