# Supplementary material for: Predicting osimertinib‐treatment outcomes through EGFR mutant‐fraction monitoring in the circulating tumor DNA of EGFR T790M‐positive patients with non‐small cell lung cancer (WJOG8815L)
Source: Mol Oncol. 2020 Nov 17;15(1):126–37. doi: 10.1002/1878-0261.12841 (PMC7782093; doi:10.1002/1878-0261.12841)
Supplement: Supplementary file 6 — Table S2. Mutations whose MFs increased at PD/stop. [file MOL2-15-126-s006.docx]

**Table S2. Mutations whose MFs increased at PD/stop**

| Assay | Gene mutation | n (%) |
| --- | --- | --- |
| ddPCR | TKI-sensitizing *EGFR* mutation | 6/43 (14.0) |
|  | *EGFR* T790M | 3/43 (7.0) |
| cobas | TKI-sensitizing *EGFR* mutation | 10/43 (23.3) |
|  | *EGFR* T790M | 4/43 (9.3) |
| NGS | TKI-sensitizing *EGFR* mutation | 8/43 (18.6) |
|  | *EGFR* T790M | 6/43 (14.0) |
|  | *TP53* mutation | 5/17 (29.4) |
|  | *PTEN* mutation | 1/1 (100) |

MF, mutation fraction
