# Supplementary material for: Predicting osimertinib‐treatment outcomes through EGFR mutant‐fraction monitoring in the circulating tumor DNA of EGFR T790M‐positive patients with non‐small cell lung cancer (WJOG8815L)
Source: Mol Oncol. 2020 Nov 17;15(1):126–37. doi: 10.1002/1878-0261.12841 (PMC7782093; doi:10.1002/1878-0261.12841)
Supplement: Supplementary file 8 — Table S4. Newly detected mutations at PD/stop. [file MOL2-15-126-s008.docx]

**Table S4.** **Newly detected mutations at PD/stop**

| Assay | Gene mutation | n |
| --- | --- | --- |
| NGS | *EGFR* C797S | 6 |
|  | *EGFR* L718Q | 1 |
|  | *BRAF* V600E | 1 |
|  | *NRAS* Q61K | 1 |

NGS, next generation sequencing
